# A hazardous substance exposure prevention rating method for intervention needs assessment and effectiveness evaluation: the Small Business Exposure Index

**DOI:** 10.1186/1476-069X-8-10

**Published:** 2009-03-26

**Authors:** Anthony D LaMontagne, Anne M Stoddard, Cora Roelofs, Grace Sembajwe, Amy L Sapp, Glorian Sorensen

**Affiliations:** 1 McCaughey Centre: VicHealth Centre for the Promotion of Mental Health & Community Wellbeing, Melbourne School of Population Health, University of Melbourne, Melbourne, VIC 3010, Australia; 2 New England Research Institutes, 9 Galen St, Watertown, MA 02472, USA; 3 Department of Work Environment, 1 University Ave, Lowell, MA 02130, USA; 4 Center for Community-Based Research, Dana-Farber Cancer Institute, 44 Binney St, Boston, MA 02115, USA; 5 Department of Society, Human Development, and Health, Harvard School of Public Health 665 Huntington Ave., Boston, MA 02115, USA

## Abstract

**Aims:**

This paper describes the refinement and adaptation to small business of a previously developed method for systematically prioritizing needs for intervention on hazardous substance exposures in manufacturing worksites, and evaluating intervention effectiveness.

**Methods:**

We developed a checklist containing six unique sets of yes/no variables organized in a 2 × 3 matrix of exposure potential versus exposure protection at three levels corresponding to a simplified hierarchy of controls: materials, processes, and human interface. Each of the six sets of indicator variables was reduced to a high/moderate/low rating. Ratings from the matrix were then combined to generate an exposure prevention 'Small Business Exposure Index' (SBEI) Summary score for each area. Reflecting the hierarchy of controls, material factors were weighted highest, followed by process, and then human interface. The checklist administered by an industrial hygienist during walk-through inspection (N = 149 manufacturing processes/areas in 25 small to medium-sized manufacturing worksites). One area or process per manufacturing department was assessed and rated. A second hygienist independently assessed 36 areas to evaluate inter-rater reliability.

**Results:**

The SBEI Summary scores indicated that exposures were well controlled in the majority of areas assessed (58% with rating of 1 or 2 on a 6-point scale), that there was some room for improvement in roughly one-third of areas (31% of areas rated 3 or 4), and that roughly 10% of the areas assessed were urgently in need of intervention (rated as 5 or 6). Inter-rater reliability of EP ratings was good to excellent (e.g., for SBEI Summary scores, weighted kappa = 0.73, 95% CI 0.52–0.93).

**Conclusion:**

The SBEI exposure prevention rating method is suitable for use in small/medium enterprises, has good discriminatory power and reliability, offers an inexpensive method for intervention needs assessment and effectiveness evaluation, and complements quantitative exposure assessment with an upstream prevention focus.

## Background

In the first National Occupational Research Agenda developed by the US National Institute for Occupational Safety & Health (NIOSH) and stakeholders in 1996, 'intervention effectiveness research' was identified as a priority area, signifying the need for expanded research efforts on how best to translate occupational health and safety (OHS) knowledge into exposure prevention and control in the workplace [1]. One decade on, there is still a need for broadly applicable methods for systematically assessing intervention needs and impacts [2,3]. Hazardous substance exposures contribute substantially to the burden of occupational disease [4]. Efficient methods for rating exposures to a broad array of substances using comparable metrics, and applying such ratings to priority setting and intervention, would be particularly useful.

Quantitative exposure or dose assessment remains the gold standard for assessing the effectiveness of interventions on hazardous substance exposures. Several considerations, however, point to the need for complementary non-analytical methods. Quantitative exposure assessment may be appropriate where one or only a few contaminants are being addressed, but is less feasible when there is a need to assess a variety of contaminants. In addition, needs assessment must take prevention and control efforts into account along with exposure levels [5]. Quantitative exposure measurements, however, do not provide information on existing control measures and do not point to upstream prevention and control alternatives. Statistical power considerations also come into play in designing intervention effectiveness evaluations. When evaluating change at the level of the work process or worksite, it is often necessary to include multiple worksites in intervention and comparison groups in order to have sufficient power to detect intervention-related change. In such cases, the need for assessing intervention effectiveness across differing sets of substances by process or worksite poses further feasibility and cost challenges to using quantitative exposure assessment.

We faced these challenges in evaluating the effectiveness of the *Wellworks-2 *intervention to reduce workplace hazardous substance exposures. This paper presents a refinement of the exposure prevention (EP) rating method developed for the *Wellworks-2 *trial, [5,6] and its refinement and adaptation for use in a subsequent intervention trial in small to medium-sized manufacturing businesses: the *Healthy Directions-Small Business *project [7-9]. This EP rating scheme was complemented by parallel evaluation with individual-level questionnaires and organizational-level assessment of OHS programs, or management systems, in both *Wellworks-2 *and *Healthy Directions-Small Business *[5,6,10,11]. The purposes of this refined EP rating scheme, in common with the first version [5], were to 1) systematically prioritize needs for intervention on hazardous substance exposures in manufacturing worksites, and 2) evaluate intervention effectiveness. The EP ratings assessed the degree of upstream prevention efforts observable in a given process or similar exposure group, consistent with the hierarchy of controls and, more recently, the precautionary principle in OHS [12-14]. This approach provides a complement to–but not a replacement for–quantitative exposure assessment. Our goal was to develop a method that could be applied with modest expense by OHS researchers and other groups engaged in workplace prevention and control efforts (e.g., independent OHS professionals, company or union OHS staff). The previous report described the theoretical basis, pilot testing and refinements, and utility for intervention needs assessment of the method [5]. In this report, we present refinements to the walk-through assessment methods, and the resulting measurement ratings and inter-rater reliability statistics for the 25 small to medium manufacturing worksites that participated in the baseline assessments of the Healthy Directions-Small Business trial.

## Methods

### Study Design and Population

The *Healthy Directions-Small Business *study was a randomized controlled trial that assessed the effectiveness of an integrated cancer prevention program. The worksite was the unit of randomization and intervention with 26 worksites recruited and pair-matched on unionization status (i.e. whether or not they were unionized). One worksite in each pair was randomly assigned to the intervention; the other to the minimal-intervention control arm. [7-9] One worksite dropped out soon after recruitment, leaving 25 worksites at the time of baseline assessments. This study was reviewed and approved by the institutional review boards of both the Dana-Farber Cancer Institute and the Harvard School of Public Health (protocol #98-333 and P10400-109 respectively), and complies with the Helsinki Declaration.

Worksites that were eligible to participate in the study had: 1) between 50 to 150 employees, 2) at least 25% of workers who were first or second generation immigrants, or people of colour, 3) less than 20% turnover rate in the past year, and 4) the ability to decide if they wanted to participate in the study (if part of a national or international company). One hundred and thirty one companies met the inclusion criteria and were invited to participate, and 26 agreed to participate. The 26 participating worksites were largely manufacturing businesses (e.g., medical equipment, dog food, specialty pumps, textiles for the automobile industry, and electronics); 3 provided laundry and printing services to other businesses. There were 96 workers per worksite, on average. More than half of the participants were white, non-Hispanic (60%), and there were significantly fewer women than men; additional demographic and description of worksites is available elsewhere [7-9].

### Healthy Directions-Small Business Intervention and Evaluation Overview

The intervention was 18 months in duration and focused on improving nutrition, physical activity, smoking cessation, and occupational health and safety. The intervention targeted behaviour change by intervening at multiple levels. These levels of intervention were: 1) individual workers (e.g., health education about diet, physical activity, smoking cessation, occupational health and safety), 2) the organization (e.g., worksite food options, lunchtime walking groups, occupational health and safety policies), and 3) the physical environment (e.g., reduction of hazardous exposures) [7,11]. The control sites received a minimal intervention of only smoking cessation programs.

### Theoretical Basis and Checklist Content

The theoretical basis of this approach has been outlined in detail previously [5]. In the "hierarchy of controls," upstream or source-focussed prevention is the most effective at exposure prevention, and downstream the least [12]. We applied a simplified hierarchy of controls to express a gradient of upstream (*materials *correspond with *source *of the hazard) versus midstream (*process *corresponds with *path *between source and worker) versus downstream (*human interface *corresponds with the level of the worker as the *receiver *of exposure) preventive efforts. This was combined with an examination of the balance between exposure potential and exposure protection at each of these three levels. The resulting Potential and Protection matrix, expressed as a 2 × 3 table, allows both a horizontal (balance of Potential and Protection at each level) and a vertical (degree to which those efforts are focused upstream) assessment of exposure prevention. Previous studies documenting upstream shifts in hazardous substance control efforts, for example in response to toxics use reduction legislation, demonstrate the feasibility of this approach as well as increasing receptivity by employers [15]. Valuing of an upstream focus is further reinforced by the precautionary principle [13,14] as well as analogous principles in other aspects of public health [16].

Six sets of indicator variables (yes/no) were developed to assess exposure Potential and Protection at the Material, Process, and Human Interface levels (detailed in Additional Files 1 and Additional file 2: the SBEI Guide and SBEI Checklist Form). Three potential routes of exposure (inhalation, dermal, ingestion) and a wide range of prevention and control–or protection–methods were assessed. Material indicators include material properties, hazard monitoring, and hazard inventory-keeping. Process level indicators include specific process types, equipment, physical conditions, and engineering and other controls. Human interface indicators include work tasks, work practices, and personal protective equipment (PPE) requirements and use.

### Data Collection

The SBEI checklist (Additional File 2) was initially written and pilot-tested in a previous study [5], and was refined for application in a small business manufacturing context for *Healthy Directions-Small Business*. All baseline walk-through assessments as well as pre-visit contacts, and site visits, were conducted by the same certified industrial hygienist (CIH), with a subset of 36 production areas also assessed by a second CIH to assess inter-rater reliability (Table 1).

**Table 1:** *Healthy Directions-Small Business *Study Site Description and Production Areas Assessed

Site Description	Employee (#)	Areas/Processes Assessed (#)	Areas/Processes Assessed for IRR (#)
**INTERVENTION SITES**			
Laundry #1	56	5	5
Food Products #1	48	7	
Electronic Instruments #1	59	5	
Fabric Finishing	121	6	
Adhesive Products	108	6	
Metal Product Fabrication #1	110	9	
Metal Product Fabrication #2	87	6	
Electronic Instruments #2	49	4	
Laundry #2	63	6	
Fabric Products #1	50	4	4
Chemical Products	86	6	
Automotive Products	64	5	5

**CONTROL SITES**			
Electronic Instruments #3	131	10	
Metal Products Fabrication #3	80	5	
Plastic Products #1	106	5	
Printing Services	32	6	6
Fabric Products #2	137	8	
Electronic Instruments #4	67	3	
Paper Products	102	6	6
Fabric Products #3	56	5	5
Metal Product Fabrication #4	76	7	
Plastic Products #2	60	8	
Food Products #2	77	8	
Metal Product Fabrication #5	115	5	5
Metal Product Fabrication #6	74	4	

Data was collected in hard copy on SBEI Checklist Forms (Appendix B) by the project industrial hygienists during walk-through inspections guided by worksite contacts. One checklist form was completed for each identifiable manufacturing process, area, or group. Groups may include maintenance departments that work in various processes or areas. In the judgment of the inspecting hygienist, processes or groups assessed on each SBEI form constituted similar exposure groups.

The first page of the SBEI checklist (Additional file 2) records general information about each process, such as numbers of workers, general air quality, housekeeping, obvious safety issues, odors, evidence of spills of potentially hazardous substances, and visible evidence of hazardous contaminants. Brief impressions of physical, safety, and ergonomic stressors are also recorded, though not incorporated into the hazardous substance exposure prevention rating process. This is followed by more specific assessments of materials used (Additional file 2, page 2), the process (Additional file 2, page 3), and the human interface (Additional file 2, page 4). Variable numbers of processes were assessed at each site, yielding a comprehensive and systematic assessment of potential for, and protection from, hazardous substance exposures for each worksite.

### Measures

Our goal in developing the following set of measures and ratings was to provide the basis for an SBEI summary score that preferentially values or rewards material-focused prevention and control, gives medium weight to process-focussed control, and values worker-focused control the least. Accordingly, materials are considered first, followed by process, and finally by human interface. Similarly, at each level (materials, process, human interface), low exposure potential was judged as more desirable than high protection from exposure.

**Figure 1:**
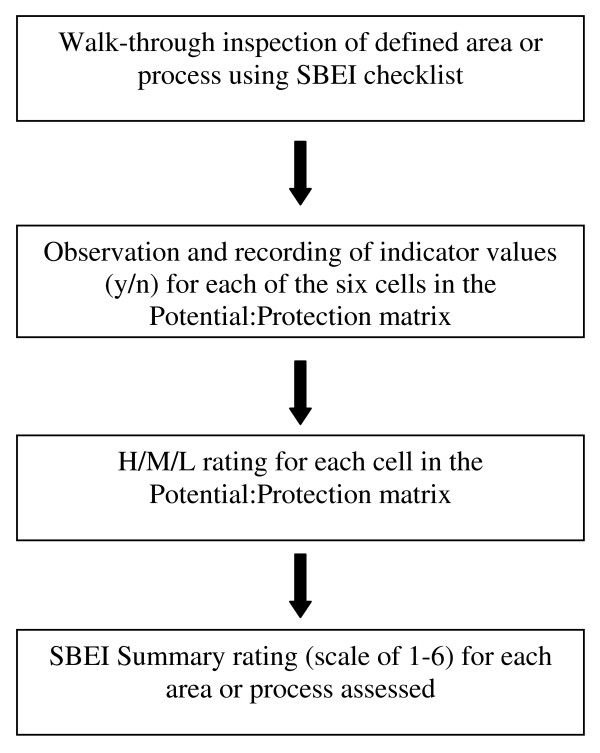


Figure 1 outlines the generation of measures from the walk-through checklist. For each set of checklist indicators (six cells of 2 × 3 matrix), a simple weighting scheme was applied wherein each indicator was designated as a Major, Moderate, or Minor contributor to potential for or protection from exposure (revised from two categories of Major/Minor in the previous version). Indicator information for each cell in the 2 × 3 matrix was then combined to give a rating of High, Moderate, or Low.

#### Material Potential

The approach to assessing Material Potential was completely reworked from the previous version, in particular to better account for concurrent Potential indicators as well as the use of multiple hazardous substances. Table 2 presents combinations of checklist indicators designated Major, Moderate, and Minor for Material Potential. Any hazardous substances in use were identified, and the checklist completed for each (see Additional file 1 for detailed guidance, and Additional File 2, page 2 of SBEI checklist). Inherent toxicity and properties, frequency of use, and quantity of use are considered in the assignment of ratings for a given substance. For example, the presence of a carcinogen, mutagen, teratogen, or asthmagen that was listed on the Massachusetts (toxic) Substance List (see Additional File 1, page 7) was deemed indicative of Major potential for exposure only if present at greater than a trace amount (> 1%) and used on a daily basis in medium to high amounts. Additional File 1, SBEI Guide, defines amount used with small corresponding to containers used on workbench, medium corresponding to drum scale, and high for large amounts used on a vat scale. Table 2 details the SBEI Checklist question numbers corresponding to these indicators. Similarly, the use of a designated skin sensitizer was only deemed indicative of Major potential for exposure if daily amount used was medium or high. The use of a carcinogen, mutagen, teratogen, or asthmagen (CMTA) present at trace amounts was considered a moderate indicator of exposure potential, as was the use of a skin sensitiser in low amounts, or high vapour pressure material (> 5 mm Hg)–not otherwise highly toxic–in medium to high amounts. Minor indicators of exposure potential were the use of high vapour pressure in low amounts, or combustion products likely or possible.

**Table 2:** Material Potential: SBEI Checklist Indicators and Area Ratings

Major Indicators*	Moderate Indicators*	Minor Indicators*	Area Rating	Count (Total = 149)
CMTA at greater than a trace amount used on a daily basis at medium to high amounts (Q1 = yes AND Q7 = yes AND Q3 = M/H;	CMTA but only at trace amount, and used on a daily basis in low amounts (Q1 = yes AND Q7 = no OR Q3 - low);	High VP material used on a daily basis in low amounts (Q4 = yes AND Q3 = L)		
Skin sensitiser and daily amount used is medium or high (Q2 = yes AND Q3 = M/H)	High VP material used in medium to high daily amounts (Q4 = yes AND Q3 = M or H);	Combustion products likely (Q5= yes);		
	Skin sensitiser used a daily basis in low amounts (Q2= yes AND Q3 = L)	Combustion products possible (Q6 = yes)		

One or more of these combinations = YES	NA	NA	HIGH	6
All NO	Two or more of these combinations = YES	NA	HIGH	6
All NO	Any one of these combinations = YES	Two or more of these combinations = YES	HIGH	3
All NO	All NO	All three of these combinations = YES	HIGH	15
All NO	Any one of these combinations = YES	All NO or one YES	MODERATE	16
All NO	All NO	Any two of these combinations = YES	MODERATE	39
All NO	All NO	Any one of these combinations = YES	LOW	12
All NO	All NO	All NO	LOW	52

*Question numbers for Additional File 1, SBEI Checklist, page 2

To assign an overall Material Potential rating (High/Moderate/Low) for a given area or process, ratings of the indicators for all materials in the area were considered collectively, as detailed in Table 2. The highest rating observed dictated the area rating. The presence of any major indicator, two moderate indicators, one moderate plus two or more minor, or three minor resulted in an area rating of High Material Potential. One moderate indicator with at most one minor indicator yielded an area rating of Moderate, as did the presence of two minor indicators. Finally, a Low Material Potential rating was assigned where one minor indicator was present, or where there were no positive indicators of Material Potential.

#### Material Protection

Indicators were also developed for Material Protection, but as in the previous version of the checklist, the main indicators were not readily or reliably observable on walk-through inspection (e.g., material inventory list maintained, MSDS available, exposure monitoring conducted)[5] Material Protection and hazard analysis were also included in the parallel organisational-level assessment of OHS programs in these sites [11]. Accordingly, Material Protection is not included in the SBEI ratings, though the indicators remain on the checklist (Additional File 2, page 2) for the reader's information.

For the other four checklist ratings, ratings for all indicators were considered collectively to assign an overall rating (High/Moderate/Low) for a given area or process, as detailed in Tables 3, 4, 5 and 6 and described in further detail below.

**Table 3:** Process Potential: SBEI Checklist Indicators and Area Ratings

Major Indicators*	Moderate Indicators*	Minor Indicators*	Area Rating	Count (Total = 149)
Q 1, 5, 6	Q 2, 3, 4, 7, 8	Q 9-18		
One or more YES	NA	NA	HIGH	46
All NO	Any 2 or more YES	NA	HIGH	7
All NO	Any 1 YES	Any YES	HIGH	31
All NO	All NO	Any 3 or more YES	HIGH	6
All NO	Any 1 YES	All NO	MODERATE	2
All NO	All NO	Any 2 YES	MODERATE	8
All NO	All NO	Any 1 YES	LOW	38
All NO	All NO	All NO	LOW	11

*Question numbers for Additional File 1, SBEI Checklist, page 3

**Table 4:** Process Protection: SBEI Checklist Indicators and Area Ratings

Major Indicators*	Moderate Indicators*	Minor Indicators*	Area Rating	Count (Total = 149)
Q 1, 2, 4	Q 3, 5, 6	Q 7, 8, 9, 10		
One or more YES	NA	NA	HIGH	36
All NO	Any 2 or more YES	NA	HIGH	19
All NO	Any 1 YES	Any YES	HIGH	32
All NO	All NO	Any 3 or more YES	HIGH	0
All NO	Any 1 YES	All NO	MODERATE	0
All NO	All NO	Any 2 YES	MODERATE	7
All NO	All NO	Any 1 YES	LOW	47
All NO	All NO	All NO	LOW	8

*Question numbers for Additional File 1, SBEI Checklist, page 3

**Table 5:** Human Interface Potential: SBEI Checklist Indicators and Area Ratings

Major Indicators*	Moderate Indicators*	Minor Indicators*	Area Rating	Count (Total = 149)
Q1, 2	Q3, 4, 5, 6	Q7, 8, 9, 10, 11, 12		
One or more YES	NA	NA	HIGH	27
All NO	Any 2 or more YES	NA	HIGH	11
All NO	Any 1 YES	Any YES	HIGH	31
All NO	All NO	Any 3 or more YES	HIGH	0
All NO	Any 1 YES	All NO	MODERATE	18
All NO	All NO	Any 2 YES	MODERATE	5
All NO	All NO	Any 1 YES	LOW	19
All NO	All NO	All NO	LOW	38

*Question numbers for Additional File 1, SBEI Checklist, page 4

**Table 6:** Human Interface Protection: SBEI Checklist Indicators and Area Ratings

Major Indicators*	Moderate Indicators*	Minor Indicators*	Area Rating	Count (Total = 149)
Q1, 2	Q 3, 4, 5	Q6, 7, 8, 9, 10, 11		
One or more YES	NA	NA	HIGH	119
All NO	Any 2 or more YES	NA	HIGH	1
All NO	Any 1 YES	Any YES	HIGH	12
All NO	All NO	Any 3 or more YES	HIGH	8
All NO	Any 1 YES	All NO	MODERATE	0
All NO	All NO	Any 2 YES	MODERATE	5
All NO	All NO	Any 1 YES	LOW	2
All NO	All NO	All NO	LOW	2

*Question numbers for Additional File 1, SBEI Checklist, page 4

Process Potential gauges how materials are being used in a given area and how likely it is that workers could be exposed because of that use. Table 3 details combinations of checklist indicators designated Major, Moderate, and Minor. Process Protection gauges observable means to mitigate or offset the potential for exposure, corresponding roughly to engineering controls. Table 4 details combinations of checklist indicators designated Major, Moderate, and Minor. Human Interface Potential gauges how likely it is that workers could come into contact with the materials being used. Table 5 details combinations of checklist indicators designated Major, Moderate, and Minor. Human Interface Protection gauges the extent to which PPE, work practices, and administrative controls are utilised to reduce exposure or contact between workers and materials. Table 6 details combinations of checklist indicators designated Major, Moderate, and Minor.

#### Small Business Exposure Index Area Summary Score

Next, we computed an overall rating of the degree of upstream exposure prevention effort for each area assessed (Figure 1, last step). The measure made use of 5 of the 6 cells in the Potential/Protection matrix. SBEI Summary scores ranged from 1 (best, minimal intervention, if any, needed) to 6 (worst, extensive intervention needed urgently). The best score was defined by low potential for toxic hazards due to the use of materials with low inherent toxicity. Where Material Potential was medium or high, but the process in which these materials were used had low potential for emissions (low Process Potential), these areas were assigned a score of 2. Where Material Potential was medium or high and Process Potential was medium or high, but this was offset by good engineering controls (Process Protection = high), these areas were assigned a score of 3. Where similar conditions to a score of 3 prevailed, but engineering controls were modest or weak (Process Protection = medium or low) and there was little potential for exposure at the Human Interface, these areas were assigned a score of 4. Where Material Potential was medium or high, Process Potential was medium or high, engineering controls were weak or modest, but effort was made to protect workers with PPE (Human Interface Protection = high), these areas were assigned a score of 5. Finally, if there was only a modest or weak level of personal protection under the other conditions prevailing for a score of 5, these areas were assigned the worst score of 6.

In short, the SBEI Summary scoring scheme cascades downstream in terms of proximity of preventive efforts to the source of the hazard. Accordingly, materials are considered first, followed by process, and finally by human interface. Similarly, at each level (materials, process, human interface), low potential was judged as more desirable than high protection.

### Evaluation of Inter-Rater Reliability

A second industrial hygienist also administered the walk-through checklist in 36 production areas across seven study sites for the purpose of evaluating inter-rater reliability (detailed in Table 1, far right column). Both hygienists were involved in the development and the writing of the SBEI Guide (Additional File 1). Assessments were conducted on the same day, within a short time of each other, such that the conditions evaluated were as close to identical as possible. The two hygienists assessed each area independently and did not communicate during assessments.

### Analysis

The individual indicator variables and ratings were tabulated over the departments assessed, with percentages reported. Although production areas were clustered within worksites, we treated the assessment of each production area as an independent measurement for these descriptive analyses.

To assess inter-rater reliability, we first computed the percent agreement for the five Potential/Protection matrix ratings (low/moderate/high), as well as for the overall summary ratings (6-point scale) for each area assessed. Weighted Kappa statistics were then calculated for each of these seven ratings. Standard arithmetic weighting was used for the six 3-point scales and the one 6-point scale evaluated. The Kappa statistic ranges from negative when the raters disagree more than would be expected by chance, to 0 when the amount of agreement is what would be expected by chance, and up to 1 when there is perfect agreement. Landis and Koch suggest the following interpretations for kappa values: *k *> 0.75, Excellent; 0.40 <= *k *<= 0.75, Good; 0 <= *k *< 0.40, Marginal.[17]

## Results

### Production Processes Assessed

The total number of production areas or processes assessed at each of the 25 worksites ranged from 3 to 10, with a median of 6 per site (Table 1). A wide variety of types of processes were assessed, including for examples: *packing and promotion *(food products), which involved the bulk transfer of material with potential airborne dust or liquid exposures; *machining *(metal product fabrication) that involved crushing, grinding, and sanding of metal parts with open tanks of fluid and the potential for airborne exposures to dusts and liquids. Some processes assessed had no observable or documented hazardous substance exposures, including for examples: *shipping and receiving in a warehouse *(plastic products), *maintenance *(printing services), and *quality control *(food products).

A wide variety of hazardous substances were captured in rated processes, including carcinogens (e.g., methylene chloride, silica, metal-working fluids), irritants (e.g., acids, nickel compounds), asphyxiants (e.g., carbon monoxide), asthmagens (e.g., epoxies), neurotoxins (e.g., methyl ethyl ketone), and reproductive hazards (e.g., lead, various chlorinated solvents such as trichloroethylene). As noted above, there were also some processes assessed where there were no observable hazardous substances.

### Potential and Protection Ratings

Material Potential ratings were generated on the basis of up to 8 hazardous substances observed in a given process. In five areas, however, there were no observable hazardous materials in use (for examples, areas described as 'Inspection' and 'Quality Control'). These areas were automatically assigned a rating of low Material Potential. Thus, one or more hazardous materials were being used in 144/149 areas assessed. In 108 areas, two hazardous materials were being used; three hazardous materials were being used in 68 areas; four hazardous materials were being used in 29 areas; five hazardous materials were being used in 9 areas; six hazardous materials were being used in 4 areas; seven hazardous materials were being used in 3 areas; and eight hazardous materials were being used in 2 areas. As detailed in Table 2, ratings of the indicators for all materials in the area were considered collectively, with the highest rating observed dictating the area rating.

For Material Potential, most areas were rated either low or moderate (80% combined) (Table 7, first row). At the Process level, the majority of areas were rated high for Potential (60%), with smaller proportions rated moderate and low (~40%) (Table 7, second row). This was offset, however, by the majority of areas being rated as having high Process Protection or engineering controls (58%). Human Interface Potential was typically high (46%) or low (38%), with relatively few areas rated moderate (15%). Human Interface Protection, or reliance on personal protective equipment, was almost always rated high (94%).

**Table 7:** Small Business Exposure Index Potential/Protection Matrix: Rating Frequencies (N = 149)

	POTENTIAL			PROTECTION		
	Rating	N	%	Rating	N	%
**Materials**	High	30	20.1			
	Moderate	55	36.9			
	Low	64	42.9			
	
**Process**	High	90	60.4	High	87	58.4
	Moderate	10	6.7	Moderate	7	4.7
	Low	49	32.9	Low	55	36.9
	
**Human Interface**	High	69	46.3	High	140	94
	Moderate	23	15.4	Moderate	5	3.4
	Low	57	38.3	Low	4	2.7

### SBEI Summary Scores

The definitions and frequencies of SBEI Summary scores are presented in Table 8. In summary, these results suggest that there was a fairly urgent need for improvements in roughly 10% of the areas assessed (scores of 5 and 6). An example in this category was the mixing oven area at a food products site that involved bulk transfer and mechanical mixing of materials (cultured whey, maltodextrin, food starches, and flavoured additives) with high potential exposures to dusts and liquid aerosols, and a reliance on personal protective equipment rather than engineering controls. There was some need for improvements in another 5% (score of 4), that there is still room for improvement–though not urgent–in roughly one on four areas (27% with score of 3). An example in this category was the soldering assembly area at a metal product fabrication site where there was potential exposure to airborne particulate not otherwise classified (NOC), inadequate local exhaust ventilation, and no respiratory protection (though general protective clothing was noted). The exposures were well controlled in the majority of areas assessed (58% of areas with scores of 2 or 1). An example in this category was the quality control (QC) process at a food products site, where there were no observable hazardous substances in use. The third column in Table 8 presents generic intervention recommendations in order of preference. These recommendations reflect the rationale of the rating scheme and encourage upstream over downstream intervention efforts, first emphasizing material factors, then process, with human interface intervention recommended only as a temporary stopgap measure.

**Table 8:** Small Business Exposure Index Area Summary Scores: Explanation and Observed Frequencies (N = 149)

Score	Definition: Explanation	Intervention Recommendations in Order of Preference	N	%
1	Material Potential low: Because the materials used have low inherent toxicity, Process Potential and Human Interface are of minimal concern.	• Minimal	64	42.9

2	Material Potential medium or high, but Process Potential low: Because there's limited potential for exposure from the process in question, then there's minimal potential for worker exposure at the Human Interface.	• Reduce Material Potential • Improve Engineering Controls	23	15.4

3	Material Potential medium or high, Process Potential medium or high, but Engineering Controls high: Material and Process Potential are significant or of concern, but well-addressed by permanent exposure controls.	• Reduce Material Potential • Reduce Process Potential	40	26.8

4	Material Potential medium or high, Process Potential medium or high, Engineering Controls low or medium, but Human Interface low: Material and Process Potential significant or of concern, but offset by low potential for exposure at the Human Interface.	• Reduce Material Potential • Reduce Process Potential • Improve Engineering Controls	7	4.7

5	Material Potential medium or high, Process Potential medium or high, Engineering Controls low or medium, Human Interface medium or high, but PPE high: Material and Process Potential significant, and matched with inadequate permanent exposure controls and an over-reliance on control at the worker through PPE.	• Reduce Material Potential • Reduce Process Potential • Improve Engineering Controls • Reduce Human Interface Potential • Rely less on PPE	14	9.4

6	All Potentials medium or high, and Engineering Controls and PPE low or medium: Exposure potential likely to be inadequately matched by protective measures.	• Reduce Material Potential • Reduce Process Potential • Improve Engineering Controls • Reduce Human Interface Potential • Rely on PPE only as a temporary stopgap measure	1	0.7

Totals:			149	100

### Inter-Rater Reliability

The percent agreement and inter-rater reliability of computed ratings are presented in Table 9. In the Potential/Protection matrix, percent agreement in subscale ratings (high/moderate/low) was high (83–89%). Summary score percent agreement was also high (78%). Correspondingly, weighted kappa statistics for subscale ratings were all in good to excellent range (0.67–0.89). Summary score inter-rater reliability was also good to excellent (0.73). The 95% confidence limits for all point estimates all excluded zero (level of agreement that would be expected by chance).

**Table 9:** Small Business Exposure Index Ratings and Summary Score: Weighted Kappa Inter-Rater Reliability Statistics (N = 36 areas)

			95% Confidence Limits	
	Percent Agreement	Weighted Kappa	Lower	Upper
	
**Potential/Protection Ratings**				
Material potential	81%	0.67	0.45	0.89
Process potential	89%	0.86	0.72	0.998
Process Protection	89%	0.89	0.78	0.99
Human Interface Potential	83%	0.80	0.64	0.96
Human Interface Protection	86%	0.73	0.51	0.96
				
**SBEI Summary Score**	78%	0.73	0.52	0.93

## Discussion

We have refined a previously developed exposure prevention rating method and adapted it to small business manufacturing settings. This rating method complements quantitative exposure assessment with a systematic and efficient assessment of prevention and control efforts with an emphasis on upstream prevention and control. It has been designed for use by researchers and evaluators as an intervention process and effectiveness evaluation tool [3]. It could also be used by practicing OHS professionals with limited budgets, which is of particular value in small business settings where OH&S resources are often very limited.

Field application of the SBEI rating method in 25 small manufacturing worksites has shown it to be capable of providing common metrics across various hazardous substance exposures found in 149 manufacturing processes or areas. Broad applicability, good discriminatory power, and excellent inter-rater reliability have been demonstrated. The main improvements over the previous (initial) version [5] were:

• Changing from two-level 'Major' and 'Minor' indicators to three levels (Major/Moderate/Minor), as detailed in Tables 2, 3, 4, 5, &6;

• Revisions to the scoring algorithm so as to better account for concurrent potential or protective factors (e.g., only rating a carcinogen as a concern if used on a daily basis in substantial amounts);

• Substantial revisions to the Material Potential assessment, in particular improvements in accounting for multiple substances being used in a single production process or area;

• A substantially expanded assessment of inter-rater reliability; and

• Full explication of scoring methods and inclusion of the actual Checklist and Guide.

Further development work is still needed. Most importantly, this includes validation of SBEI scores against quantitative and other exposure metrics.

The data gathered might be improved by additional interview of the site staff person guiding the walk-through assessment, line supervisors, and workers in the area. While such interviews would surely provide a deeper and broader assessment, we anticipated that this would not be feasible in most study sites due to a combination of production pressures, the sensitivity of OHS issues in many workplaces, and other concerns. Other concerns include the challenge of reliably interviewing workers in private while being guided by someone who is usually a management representative (in order to get frank responses and data of comparable quality across all areas assessed), and how to combine data in situations where different interviewees respond differently to the same or similar questions. In summary, we believe that incorporating interviews of walk-through guide, line supervisors, and workers would overly complicate the administration of the SBEI checklist. This would also represent a different measure–one based on site employee perceptions rather than an outside industrial hygienist. Our strategy in the *Healthy Directions-Small Business *study as a whole has been to gather data on worker perspectives (through confidential surveys at individual worker level) and OHS programs (organizational level) separately and in parallel to the SBEI assessments (physical environment level) [6]. Taken together, these three levels provide a comprehensive assessment of OHS conditions for both needs assessment and evaluation purposes. Cross-comparison of OHS performance across these levels and measures could be the subject of further analyses of these data (e.g., to demonstrate convergent validity). Separately, these three levels each provide a different way of understanding OHS hazards or exposures. The SBEI assessment discussed in this paper provides a low-cost, feasible method by which to independently evaluate hazardous substance exposures and prioritize interventions.

### SBEI Scores

The distributions of ratings showed reasonable discriminatory power of the SBEI exposure prevention rating method, with a general pattern towards low Potential and high Protection ratings, and a distribution of overall SBEI scores that was strongly skewed towards the favourable end. A similar pattern was observed previously in large manufacturing worksites in the *Wellworks-2 *trial [5]. The frequency of favourable ratings in our sample may be artefactually elevated relative to the full population of manufacturing worksites due to the selection biases inherent in this study. Participating companies had to voluntarily agree to occupational health intervention together with health promotion if they were randomized to the integrated intervention group [7]. Thus companies that have exposure concerns or that do not place a high priority on occupational health would have been less likely to participate. We would also note, however, that many companies that expressed willingness to participate noted the OHS consultations as an important incentive.

Despite the likely overestimate of favourable ratings in comparison to the full population of small manufacturing businesses, a gradient of intervention needs was identified in our sample. Significant fractions of the sample received the very poor (~10% with score of 5 or 6) or intermediate (~32% with score of 3 or 4) SBEI scores. A strength of these scores is that each has corresponding intervention recommendations to guide the user in shifting prevention and control efforts upstream. In this regard, the detailed Potential/Protection matrix and SBEI scores perform a detailed needs assessment and prioritization function as well as providing baseline measures for effectiveness evaluation. As noted in the Introduction section above and elsewhere [18], occupational disease constitutes a substantial and inequitably distributed burden. Thus prioritising of intervention using the SBEI approach, which in this sample identified 10% in need or urgent attention, represents an expeditious and targeted means to reduce this burden.

### Inter-Rater Reliability

Inter-rater reliability of the 5 Potential and Protection ratings used to compute SBEI ratings was good to excellent, and the overall SBEI exposure prevention summary scores demonstrated the best reliability of all. Because the two observers were both involved in instrument and protocol development, however, this may overestimate the inter-rater reliability that would be observed with two completely independent reviewers working solely from the written protocol. This limitation notwithstanding, field performance of the SBEI scoring method is good to excellent.

### Validity

The basis of the SBEI scoring method on the hierarchy of controls supports its face validity. Furthermore, when used as pre- and post- intervention effectiveness measures as intended in this study, the baseline assessment of each area serves as its own reference or control, with the final evaluation metric being a measure of change [6]. To the extent that a given area or process does not change fundamentally over the course of the intervention (e.g., gets replaced with an unrelated process or gets phased out), this strategy overcomes limitations inherent in comparing area ratings and scores cross-sectionally as well as longitudinally (as an intervention effectiveness measure).

We would hypothesize that cross-comparison of ratings and scores across production areas assessed and study sites would show corresponding relative levels of hazardous substance exposures. This has not been assessed in the current study because of the developmental stage of the instrument, technical and economic feasibility issues, and concerns about decreasing participation. With respect to feasibility, numerous agents would have to be sampled many times in each area assessed, which would involve considerable expense. In addition, requests to conduct such extensive sampling in the recruitment phase would be likely to further bias the sample of participating companies towards those with relatively good exposure control programs.

One approach to validation would be to obtain summary measures from multiple quantitative exposure measurements for each hazardous material in each area assessed. Measurements for each agent could then be transformed to a percent of a chosen set of Occupational Exposure Limits (OEL) (e.g., ACGIH, NIOSH, or OSHA). These summary percent OEL's could be averaged into an overall percent OEL across the range of agents present in each given area, paired with SBEI scores for each area, and analysed using standard correlational methods. We explored this possibility in the *Wellworks-2 *study, but found that routine monitoring was reported for only 14% of areas assessed [5]. This showed that there was not enough company-collected quantitative exposure data available for validation studies even in large manufacturing worksites. We expected even less routine exposure monitoring to occur in small manufacturing settings, and thus did not explore this possibility in the *Healthy Directions-Small Business *study. Additionally, this demonstrates a gap in workplace exposure assessment practice that might be addressed in part through the application of more economical alternative strategies such as the approach described in this report.

### Comparison of SBEI to Other Exposure Rating Schemes

Comparable assessment approaches to other hazardous exposures may also be feasible, such as ergonomic, safety, or other hazards. The development of a similar health and safety rating system has been reported for farm operations, wherein 'positive aspects' are balanced against 'negative aspects' for four different farm characteristics (operator attitude, operator characteristics, status of facility, and status of equipment) [19]. A Site Rank Score is generated as the average ranking of the four characteristics. In this example, a very similar conceptual approach to the SBEI was generated independently for a different work context. Such rating schemes have broad applicability beyond manufacturing work settings.

SBEI also shares fundamental characteristics with control banding. The control banding approach has evolved from its origins in the pharmaceutical industry [20] to the sophisticated yet accessible web-based "COSHH Essentials" program designed by the U.K.'s Health and Safety Executive to assist small businesses in their efforts to understand and control risk http://www.coshh-essentials.org.uk[21]. Control banding allows managers to use process knowledge, walkthroughs, toxicological data and other information sources to assign a job task to a "control band" based on risk potential. The band dictates the level of control needed for a particular operation. SBEI similarly promotes the integration of multiple hazard and exposure information sources, but also incorporates into its assessment the current control strategies for each operation. SBEI extends the control banding approach to create an integrated framework of risk and control assessment. The SBEI measures also extend control banding approaches in providing ratings and scores that are usable as intervention effectiveness evaluation measures.

## Conclusion

The SBEI scoring method shows great promise as a new tool for interventionists and intervention researchers alike, fulfilling both needs assessment and evaluation functions. Most importantly, this systematic approach complements quantitative exposure assessment with its focus on assessing preventive efforts rather than the downstream phenomenon of worker exposure. The method guides and directs the user toward upstream prevention solutions to common hazardous substance exposure issues, encouraging prevention- over control-oriented occupational health practice in the workplace. We hope that open access to the SBEI checklist and guidance materials (see Additional files) will foster its adaptation and use by others.

## Abbreviations

ACGIH: American Conference of Government Industrial Hygienists; CIH: Certified Industrial Hygienist; CMTA: Carcinogen, Mutagen, Teratogen, or Asthmagen; COSHH: Control of Substance Hazardous to Health; EP: Exposure Prevention; IRR: Inter-Rater Reliability; NIOSH: National Institute for Occupational Safety and Health; NOC: Not Otherwise Classified; OEL: Occupational Exposure Limits; OHS: Occupational Health and Safety; OSHA: Occupational Safety and Health Administration; PPE: Personal protective equipment; SBEI: Small Business Exposure Index.

## Competing interests

The authors declare that they have no competing interests.

## Authors' contributions

ADL, CR, and AMS conceived of and revised the content and scoring of the Small Business Exposure Index. AMS led data management and statistical analyses, with contributions to the development of scoring algorithms from all co-authors. GS led the *Healthy Directions-Small Business *study, as well as participating in the development of the SBEI. ADL led the writing of the paper. All authors reviewed and approved the final version.

## Supplementary Material

Additional file 1 Small Business Exposure Index GUIDE. Click here for file

Additional file 2 Small Business Exposure Index Checklist Form. Click here for file
