# Supplementary material for: A hazardous substance exposure prevention rating method for intervention needs assessment and effectiveness evaluation: the Small Business Exposure Index
Source: Environ Health. 2009 Mar 26;8:10. doi: 10.1186/1476-069X-8-10 (PMC2679725; doi:10.1186/1476-069X-8-10)
Supplement: Additional file 1 — Small Business Exposure Index GUIDE. [file 1476-069X-8-10-S1.pdf]

<u>Small Business Exposure Index GUIDE</u>

Additional File 1, published with:
LaMontagne et al (2009): A hazardous substance exposure prevention rating method for intervention needs assessment and effectiveness evaluation: the Small Business Exposure Index. *Environ Health.*

This guide is intended to assist the rater in filling out the Small Business Exposure Index checklist form. This form is intended to rate defined areas in each of the recruited worksites in the project, at the start of the project (baseline) and again at the conclusion of the project (final). Although this is the primary purpose of the form and rating procedure, it is also hoped that the process described can be a useful, proactive (prevention oriented) recognition and needs assessment tool for general workplace health and safety and industrial hygiene applications. The method provides a conceptual and systematic process for examining a workplace, particularly during the initial assessment.

The process divides the workplace into logical divisions with a goal of defining "potential" and "protection" for uniform exposure groups. The process evaluates the defined area / group in a general way (**page 1**) followed by an assessment of potential for exposure and protection from exposure in each of three major areas: material (**page 2**), process (**page 3**) and human interface (**page 4**), Detailed instructions follow:

(**NOTES:** Throughout the form, items in ***bold type*** will likely require discussion with management to be answered/assessed.
Score 1 point for each question answered" yes" unless there are more specific instructions listed in the GUIDE.)

<u>PAGE 1: GENERAL</u>

<u>Defined Group:</u> This will almost always be a specific area at the site(exceptions might be maintenance or a transportation group that moves around the site).
In addition to the goal of "<u>similar potential chemical exposure</u> " other characteristics might include: logical and definable, convenient size and location, and a group where intervention effects can be seen.

2. **<u>Other Processes:</u>** The Defined Group is likely to involve only one major process. Where this is the case, this question provides space to list secondary processes and the # of employees associated with each. If there is more than one <u>major</u> process, consider defining that as a separate Defined Group.

3.List the **total # of employees** on all shifts in the workplace and the number of employees on all shifts in the Defined Group.

4. **Work Density** is a measure of activity in the worksite and the proximity of one worker to the materials being used by other workers. Estimate the square footage of the area occupied by the Defined Group and divide by the number of employees in the group.

(***NOTE:*** **Questions 5 –14 below refer to the general area being assessed – the Defined Group/Area – with an emphasis on the *major process* in the area. In other words, hazards and conditions related to other processes (See Question 2 above) in the area may be included unless there is a difference between the hazard and condition in the *major process* and the *other process*. For example , if the ceiling height, odor, or housekeeping rating/assessment was different between the two processes, choose the rating for the major process.)**

5. **Building**: Estimate the ceiling height of the Defined Area. Circle all applicable conditions which may add to potential exposure concerns (not related to production).

6**. Air:** Check this item if there are visible materials in the air associated with the process or in the work area.

7**. Surfaces:** Check this item if there are visible materials on work surfaces which could become airborne or to which employees could come in contact.

8. **Odors:** Check if there are significant odors present (that might indicate exposure).*Do not* checks if odor is faint or barely detectable.

9**. Leaks, Spills**: Check if leak or spill is recent or continuing. *Do not* check for old stains or evidence of past leaks that have been corrected or cleaned up.

10. **Housekeeping:** Good housekeeping has often been characterized as "a place for everything and everything in its place". Bad housekeeping can then be seen as the opposite: no designated place for everything and everything not in its place. Good housekeeping is more than tidiness, however, and often has a direct bearing on health (exposure to dangerous contaminants) and safety (hazards that lead to serious injuries). Good housekeeping involves both design

and practice; personnel, policies, procedures and equipment as well as actually practicing good housekeeping as part of the job. A five point scale to characterize good and bad housekeeping is described below. The general approach for this rating will be a two step process: (1) focus on “acceptable” housekeeping as described and decide whether the observed situation is acceptable, good (better) or bad (worse). (2) If good or bad is chosen, decide whether or not the situation is very good or very bad.

Acceptable Housekeeping: The area is **generally clean**, clear of debris or obstructions with **few** loose or **out of place** tools, materials or other items. Aisles are marked and generally clear, trash containers are available and few if any housekeeping related **hazards** are observed (e.g. slippery floors, improper storage, tripping hazards, obstructed exits/safety equipment, etc.)

Conditions that are better (good)or worse (bad) than this norm would then be determined as follows:

Good housekeeping: The area is clean, clear, and free of debris or obstructions**. No** tools, materials, or other items are **out of place** and no housekeeping related hazards are observed. *(Score = -1)*

Bad housekeeping: Compared to acceptable, the area is **unclean** and cluttered, with tools, materials and other items out of place. Housekeeping related **hazards** are observed.

Having determined that housekeeping is good or bad, compared to acceptable, the next step involves deciding whether housekeeping is very good or very bad. This determination may go beyond observation of the area and involves planning, organizing and designing for good housekeeping. Examples to illustrate this point: (1) drip pans under leaking machinery or containers under leaking spigots may indicate good housekeeping, but policies and procedures to fix leaks or clean up spills in a timely manner is planning and designing for good housekeeping. (2) In a similar way tools kept together in a box indicates good housekeeping practice, but a peg board with tool outlines goes beyond neatness to a system that encourages good housekeeping and good work practices.

Very Good housekeeping: The area is exceptionally clean, uncluttered/and orderly. There is **evidence** of splash guards and other **controls** to prevent dispersion of contaminants. Employees in the area not only work in a way that keeps the area clean, but also have **time for regular clean up**. There is evidence of periodic cleaning of walls, windows, light fixtures, rafters and machinery.

Very Bad housekeeping: The area is exceptionally dirty, cluttered and **disorderly.** There is little evidence of preventive practices, good housekeeping design or policies/procedures to encourage good housekeeping. There may be

evidence of spills (not cleaned up immediately), as well as dirty windows, light fixtures and **rafters.**

11**. Air Quality**: Air quality is a subjective condition which may change dramatically from day to day, from initial entry to longer stay (olfactory fatigue), from individual to individual, and may depend on the weather. Although these limitations exist for outside observers coming into the area as well as employees, the following guidelines are described for the five point air quality scale used in this instrument (and presented on the employee survey). Air quality will be determined with a two step process: (1) is the air quality acceptable, good (better) or bad (worse)? (2) If the air quality is good or bad, compared to acceptable, is it very good or very bad?

Air quality will be determined primarily by observation with additional investigations conducted if necessary. Observation will include:

- Initial impressions including odor and general comfort (temperature, humidity, air flow).
- Visible clues
  - Smoke, mist, fumes from operations or outside sources
  - Dust or liquid on surfaces
  - Staining or evidence of past water leakage or chemical spills
- Irritation of eyes, throat, skin
- Employee complaints or use of respiratory protection
- Engineering controls in place

Acceptable air quality: The areas are generally **comfortable** (temperature, humidity, drafts) with slight or no odors and no irritation. There are few to no visible clues in terms of visible emissions, material on surfaces and evidence of leakage or spills.

Good air quality is defined as very comfortable conditions with **no odors** and no irritation. There are **no visible** emissions, contaminants on surfaces or evidence of leakage/spills.

Bad air quality is defined as conditions with **comfort issues** (temperature, humidity, drafts) where **odors** and/or irritation (eyes, throat, skin) may be present. There will likely be visible emissions, contaminants on surfaces and/or evidence of leakage or spills.

Very Good air quality: This rating will take into account the comfort, odor and irritation factors described in “Good”, but will also include factors that keep the air quality at this level. For example, **engineering controls** in place that are properly designed and operated, adequate make up air systems and periodic inspections and/or measurements to check the operation of LEV and HVAC systems.

Very Bad air quality: This rating will take into account the comfort, odor and irritation factors described in “Bad”, but will also include evidence of these issues among employees. For example, have there been **complaints** of discomfort, irritation or other health effects? Are there employees that regularly use **respiratory equipment?** (required or voluntary)

12**. Physical Stressors**: Describe the physical hazards / exposures in the Defined Area by circling appropriate words and adding the # of employees exposed in the space provided. Note location or other information on the line below the list of stressors.

- heat: means high temperature from work processes *not* seasonal heat waves

- cold: as part of the process or job, for example, working outside or near a cold process. Lack of heat in an area should be covered in #5, BUILDING.

- noise: check if estimated noise levels are greater than 85dbA, even for short term exposures.

- radiation, including ionizing or non ionizing forms as part of the process or job.

- Lighting : is there sufficient illumination, is emergency lighting present (if necessary)

13**. Safety hazards** : Describe the safety hazards in the Defined Area by circling the appropriate words and adding the number of employees exposed to that hazard in the space provided. Note location or other information on the line provided.

- fire includes hazards associated with fire and flammable materials : portable extinguishers and other fire protection equipment (sprinklers, fire doors) present & maintained; flammable storage limits; storage cabinets; flammable waste; fire brigades; etc…….

- elect includes electrical hazards: control panel id's, grounding and bonding, exposed conductors, lock out-tag out…….

- W/W means walking and working surface hazards : slip, trip and fall hazards, aisle marking, aisle clearance, platforms/scaffolding, ladders, stairs, exits, material handling……..

- guarding : for mechanical, hydraulic and pneumatic power systems; pinch points, nip points; guards secured and properly adjusted……..

- gas means hazards associated with compressed gas cylinders: storage, marking, segregation, caps in place……

14. **Ergonomic stressors**: Describe the ergonomic conditions in the Defined Area including # of employees exposed.

- rep / motion : repetitive motion is part of the job

- ex/force : excessive force (for example, pinch grips, hand hammering) is part of the job

- awk/pos : there are ergonomic concerns about the workstation or work position that result in awkward postures.

- incentive/rest : work involves piece work pay system and/or inadequate rest breaks

- mach / pacing : job is paced by an assembly line or machine

- tools : there are ergonomic concerns about the tools used (handle size, vibration, weight, etc.)

- lift / move heavy : job involves lifting and/or moving heavy parts or material.

- <u>seat / bench</u> : concerns about the seat or workbench (no adjustment, four legs etc.)

- <u>shoulder / knee</u> : job involves lifting or handling materials below knees and / or above shoulders

PAGE 2: MATERIAL

(Material) Instructions

An obvious aspect of any exposure situation is what materials are involved and how toxic are those materials. Toxicity, the amount of material used, form, and possible decomposition / combustion products represent the *potential* for harm if there is exposure*. Hazard Analysis* refers to those actions which inform users of the materials about the potential for harm and the procedures / equipment that may be in place to provide protection

The investigation starts with a listing of the materials used in the Defined Area and an examination of MSDS's for those materials. Although an area specific collection of MSDS's may exist it may be necessary to interview management representatives or directly observe the work in order to determine the materials used. List the described information for each material on the lines provided on the form. In some cases, for example maintenance, it may make sense to list "categories" of materials used like welding rods or lubricating oils instead of each individual (and very similar) item.

(Note: If there are more than four significant materials (or categories) in use in the area, use another copy of page 2 and continue the letter designations A = E, B = F, etc)

Evaluate each material with respect to the "potential" and "protection" questions by putting a check, or appropriate letter, in each box if the answer is yes.

1. Using the Massachusetts Substance List (produced by the Mass. DPH Right To Know Program, 1993 version, and/or the MSDS for the material) does the material contain any ingredient designated as a **<u>Carcinogen, Mutagen,</u> <u>Dermatogens</u> <u>or Asthma</u> <u>Producing</u>.** Put the appropriate letter(s) in the block.

2. Same as above: **<u>S</u>** = skin designation, **<u>SS</u>** = skin sensitizer.

3. Make an estimate of the **daily amount** of the material that is being used;

- L is low or **"bench"** amount , e.g. small containers of the material stored and/or used on the workbench.

- M is moderate or **"drum"** amount , e.g. more than bench amount used and facility stores or purchases material in bulk amounts, e.g. "55 gal drums"

- H is high or **"vat"** amount, e.g. large amounts of material are used in Tanks or large containers, vats, in the work area.

4. Put check in box if **vapor pressure** is greater than 5mm Hg.

5. Answer yes if there is the likelihood that **combustion/ decomposition** could take place as a result of the **process**, e.g. overheating, runaway reaction, etc.

6. Yes, if hazardous **combustion/ decomposition** products are *possible* based on information from the **MSDS.**

7 Although it is important to know if *any* Low / No Threshold substances are in the material, more than a **trace amount** (1% or less) is obviously significant.

Hazard Analysis

1. Self explanatory

2. Self explanatory

3. Self explanatory

4. Has the **hazard assessment** required by OSHA's revised PPE standard been done?

5. Is **air sampling** with any type of sampling equipment done routinely ? Are ventilation systems (necessary to control exposure) routinely monitored for **performance**?

6. Has the activity in #5 **ever** been done.

7. If, based on MSDS information, an **eyewash/shower** is necessary, is an acceptable unit in place. (Note: it is unlikely that squeeze bottles will ever be acceptable)

8. Self explanatory

9. This includes both **labels/warnings** on containers (as required by Haz Com), as well as necessary signage required by the use of the particular materials, e.g. if ppe is called for by the MSDS, are there signs to remind/alert people to this need?

PAGE 3: PROCESS

(process) Instructions

The next area being investigated and evaluated is process; how is the material (regardless of how toxic it may be) being used and how likely is it that workers will be exposed because of that use? and… What kinds of process protections, engineering controls, are in place to mitigate or balance the potential for exposure. Process items are often easier to see and identify because they are usually visible even if the process is not operating.

Notes:

(a) Where appropriate, circle the specific operation observed.

(b) The items here generally apply to the process and job that is being done. Where the process also involves maintenance operations, the situations can be confusing. For the purposes of our form, if maintenance type operations – cleaning, adding chemicals, adjustments – are done as part of his/her job, they are covered here. If these operations are done by another person, they should be covered under that group.)

1. Spraying as a **primary activity** means spraying is specifically part of the process.

2. **Visible mist or spray** in contrast with #1, indicates the *escape* of mist/spray from the process.

3. **Transfer of material:** Exposures are likely to happen at the places in the process where bulk material is added to the process, transferred from container to container, or put in containers for shipment. Check if there are material transfers and the opportunity for exposure.

4. Is **abrasive blasting** of any kind being done including " glove box " operations

5. Circle which of these **fume-producing** operations is being done.

6. Circle which of these **dust-producing** operations is being done.

7. Check if the operation is **plating** or other similar operation.

8. This question refers to **elevated (process) temperature** ; if ambient temperature is *also* an issue, it is covered on Page 1, #12.

9. **Open tanks or containers** that are part of the process, they are not just open because the covers have been left off. This item *should* be checked for electroplating operations.

10. Is **mixing** being done in such a way that there could be exposure to dust or liquid e.g. open vessel or no cover on mixer?

11. Is any **molten metal** involved in the process even if temperatures are not high enough to produce fume (see item 5 above)?

12. Is there a **release of particulate** material (dust, mist) that would not be covered by other categories? (NOC= Not Otherwise Classified)

13. Circle the type of **machine operation** being used. (EDM= Electro Discharge Machining)

14. Operations involving **plastic molding , extrusion** or similar heated deformation of the material are covered here.

15. Materials in a gaseous form are used in the process, e.g. “dopant” gases, gas for heat treating atmospheres.

16. Does any part of the process involve **pressurized containers** of gas or liquid?

17. Does any part of the process involve the **drying of parts** that have been wetted with liquid, for example, painting or vapor degreasing.

18. If there are **other** aspects of process potential that are *not* covered above, describe the aspect and check the yes box.

Protection / Engineering Controls

1. **Totally automated** means that virtually all aspects of the *operation* are *done by machine*; for example, only the finished product is handled as it exits the machine (s).

2. **Totally enclosed** means that significant *protection* for the operator is provided by the barriers; for example, the operator might interact or handle the product at the beginning and/or at the end of the process, but the barriers provide separation and/or prevent process materials from contacting the operator. In many instances a totally automated process will also be totally enclosed, but there is a distinction between “done by machine” and “protection”.

3. **Local Exhaust Ventilation** (LEV) is provided, is working and *visually* appears to be properly sized and designed.

4. **Operator enclosure** is an alternative control method to # 1 and # 2 above and means that the “control” focuses on the operator instead of the process.

5. **Semi – automated** means that the operator must interact with or do some part of the operation being performed. From another perspective, the machine does only part of the operation.

6. **Partially-enclosed** refers to those situations where the enclosure or partial enclosure provides *some* protection as contrasted with *significant* protection in # 2.

7. **HVAC** (Heating, Ventilation, Air Conditioning) and/or **general dilution ventilation** (e.g. ceiling fan) is present and in operation (working)..

8. **LEV is present** but there may be questions about the adequacy of the system (design, sizing, maintenance, etc.) Do not answer if #3 was answered yes.

9. Check yes if **make up air** is adequate.

10. **(other)** Add items that provide protection, that are engineering controls and that are not listed above.

PAGE 4: HUMAN INTERFACE

Having described the materials being used and the process being done, the third major area is describing what the operator may do as part of his/her job that will increase the likelihood of being exposed (Potential for exposure) and…..what the operator does (including wearing PPE) that would decrease the likelihood of exposure (Protection from exposure).

Human Interface: Potential

!. Any **application** of material such as painting, gluing, assembly, etc.

2. Does any part of the job done by people in this *defined group*, involve **mixing, adding or stirring** chemicals?

3. Is **compressed air** used for cleaning the workstation or for the process, e.g., blowing liquid off parts?

4. Self explanatory

5. Check yes if the material(s) used, like lead or cadmium, may include **ingestion** as a major route of exposure.

6, Is the work done in such a way that **exposure is more likely** when the job *could be done differently*, e.g. bending over an open container or working in the plume of the welding arc?

7. This item may be confused with #1 & #2 above, but is intended to **emphasize the manual** aspect. For example, is the job being done manually when mechanical means could be used?

8. This is a measure of how hard someone is working to do the job. **Speed, repetition and/or heavy lifting** could contribute to this answer.

9. Self explanatory

10. The work surfaces have **materials** of concern that may be **contacted** in the normal course of work.

11. Check yes if **manual cleaning** is part of the process and the operator's job.

12. Add **other** item(s) not covered in the above questions.

Human Interface: Protection/PPE

1. **Respirators**, including dust masks, are **required** to be worn and are being worn.

2. **PPE** is **required** and is being worn. Circle or name the PPE items required and worn.

3. The process has been designed or steps have been taken to minimize or decrease the amount of **<u>material handling</u>**, for example, automation or a specific process change.

4. **<u>Work practice</u>** or how the job is being done makes it less likely that exposure will occur as compared to *other ways* the job could be done. For example, adding chemicals slowly and close to the container to avoid splashing.

5. Some form of **<u>administrative control or job rotation</u>** is being used to decrease exposure on the job.

6. **<u>Respirator</u>**s, including dust masks, are being **<u>used</u>** but are not required. Do not answer yes if #1 is yes.

7. Protective clothing and equipment is **<u>available and appropriate</u>** for the hazard(s), even though it is not being used. Do not answer yes if #2 is yes.

8. **<u>Nearby</u>** means close enough to be used frequently if needed. Most of the time this would mean *in the work area.*

9. This item refers to an area/room that has been set aside for **<u>eating/breaks</u>** that is kept reasonably free from workplace contaminants.

10. Is the **<u>eyewash/shower</u>** present in the area (See Material: Protection # 7) adequate: working, maintained, not obstructed….In almost all cases , the small mounted squeeze bottles will not be considered adequate.

11. Add <u>other</u> protection items not covered above.
