# Supplementary material for: A hazardous substance exposure prevention rating method for intervention needs assessment and effectiveness evaluation: the Small Business Exposure Index
Source: Environ Health. 2009 Mar 26;8:10. doi: 10.1186/1476-069X-8-10 (PMC2679725; doi:10.1186/1476-069X-8-10)
Supplement: Additional file 2 — Small Business Exposure Index Checklist Form. [file 1476-069X-8-10-S2.pdf]

SMALL BUSINESS EXPOSURE INDEX

LaMontagne et al (in review): An Exposure Prevention Rating Method for Intervention Needs Assessment and Effectiveness Evaluation: the SBEI

SITE NAME: ________________________ DATE ___________

ACCOMPANIED BY: ___________________________ TITLE: _________________________

1. DEFINED GROUP w/ similar potential chemical exposure (choose by ***area, process, product, dept***,…)____
   ____

2. OTHER PROCESSES in area & (#employees at each)______________________________
   ____

3. ***No. of employees*** (all shifts) GROUP_________________& TOTAL ________________

4. WORKER DENSITY (sq. ft Area per EE) ____

5. BUILDING characteristics/conditions: CEILING height______________________________
   (Incidental potential exposure NOT related to production)
   circle: dampness, ***pest control, renovations,*** painting, other___________________________

	YES	NO	DESCRIBE
6. Contaminants visible in the AIR (dust, mist, process plume)?	☐	☐	________________
7. Contaminants visible on SURFACES (dust, grit, film, liquid)?	☐	☐	________________
8. Mod/strong ODORS detectable in the area?	☐	☐	________________
9. Indications of recent/on-going LEAKS or SPILLS in the area?	☐	☐	________________

10. HOUSEKEEPING in the area is:		11. Overall AIR QUALITY in the area is:	
Very Good (***system, time***)	☐	Very Good (eng. controls)	☐
Good (no out of place)	☐	Good (no odor, visible)	☐
Acceptable (gen. clean, few)	☐	Acceptable (comfortable)	☐
Bad (hazards, dirty)	☐	Bad (discomfort, odor)	☐
Very Bad (long time)	☐	Very Bad (ppe, ***complaints***)	☐

	YES	NO	N/A	Describe
12. Are employees potentially exposed to PHYSICAL stressors?	☐	☐	☐	_______________

If yes, circle+(#ee's): heat( ) cold( ) noise( ) radiation( ) lighting( ) other( )_____________
____

	YES	NO	N/A	Describe
13. Are employees exposed to SAFETY hazards?	☐	☐	☐	_______________

If yes, circle+(#ee's): fire( )elect( ) w/w( ) guarding( ) gas( ) other( )_________________
____

	YES	NO	N/A	Describe
14. Are employees exposed to ERGONOMIC stressors?	☐	☐	☐	_______________

If yes, circle+(#ee's): rep/motion( ) ex/force( ) awk/pos( ) ***incentive/rest***( ) mach/pacing( ) tools( ) floor( ) lift/move/heavy( ) bench/seat( ) shoulder/knee( ) other( )_____________
____

Areas/issues requiring further explanation:

MATERIAL

Material Potential: Looking at the characteristics of the materials, how hazardous are they and how much and in what form are they used?
Hazard Analysis: To what extent are there hazard analysis procedures in place to minimize the *Material Potential*? (Note: This category may overlap with the H&S Program Evaluation form)

For this section, review the materials used by the defined group (see #1) by: ***reviewing area specific MSDS's, conducting management Interviews*** and/or direct observation of the work. (Note: list materials from area and review MSDS's later for specific hazard info.)

MATERIALS USED (1:common name/tradename, 2:principle ingredients & %, 3:Form: S,L,G)

A.____

____

B. ____

____

C.____

____

D.____

____

**POTENTIAL**	Materials			
MATERIAL POTENTIAL:	A	B	C	D
(Major)<br>1. Contains Low, No threshold materials<br>(C = carc, M = muta, T = terat, A = asthma)				
2. Skin sensitizer(SS) / skin designation(S)				
3. ***Daily amt. used*** (L=bench, M=drum, H=vat)				
(Minor)				
4. High vapor pressure (> 5mm Hg)				
5. Combustion/decomp. prod. likely (process)				
6. Combustion/decomp. prod. possible (MSDS)				
7. More than trace amount of #1				

**PROTECTION**<br>HAZARD ANALYSIS:				
(Major)<br>1. ***Material inventory maintained (list)***				
2. MSDS's present in defined group (delete) ---	-------------	--------------	-------------	------------
3. MSDS's available, all shifts				
4. Hazard Assessment done (OSHA PPE)				
5. ***Monitoring routinely done***				
(Minor)<br>6***. Monitoring (sampling) ever done***				
7. Eyewash / shower present (if needed)				
8***. Chem. emergency plan posted/available***				
9.. Proper signage present (labels, warnings)				

Comments:

PROCESS

Process Potential: Looking at the characteristics of the process, how likely is it that exposure could occur?
Engineering Controls: To what extent have ventilation and process controls been put in place to decrease the *Process Potential*?

**POTENTIAL**				
PROCESS POTENTIAL:				
(Major)<br>Process involves(circle specific item):	Yes	No	Don't Know	Describe
1. Spraying as primary activity (painting/coating)	☐	☐	☐	
2. Visible mist or spray, e.g. as byproduct	☐	☐	☐	
3. Bulk ***transfer of material*** (pot/airborne)	☐	☐	☐	
4. Abrasive blasting (inc. small cabinets)	☐	☐	☐	
5. Welding, brazing, flame/arc cutting/spraying	☐	☐	☐	
6. Crushing, sanding, grinding, buffing (circle)	☐	☐	☐	
7. Electroplating operations	☐	☐	☐	
8. Elevated temperatures (***>> ambient***)	☐	☐	☐	
(Minor)<br>Process & job(not ***maintenance***) involves:				
9. Open tanks or containers(not housekeeping)	☐	☐	☐	
10. Mechanical mixing (dust/liquid = exposure)	☐	☐	☐	
11. Molten metal , e.g. solder pots, casting	☐	☐	☐	
12. Release of particulates (NOC)	☐	☐	☐	
13. Machining: lathe, drill, mill, EDM, other:	☐	☐	☐	
14. Plastic molding operations/extrusion	☐	☐	☐	
15. Materials in gaseous form	☐	☐	☐	
16. ***Elevated pressure***, part of process	☐	☐	☐	
17. Drying of liquid covered parts	☐	☐	☐	
18. ***Other process element***	☐	☐	☐	
**PROTECTION**				
ENGINEERING CONTROLS:				
(Major)				
1. Process totally automated (finished part out)	☐	☐	☐	
2. Process totally enclosed (product in/out only)	☐	☐	☐	
3. LEV – appropriate and ***working***	☐	☐	☐	
4. Operator totally enclosed or separated	☐	☐	☐	
(Minor)				
5. Process semi-automated (***some oper. work***)	☐	☐	☐	
6. Process partially enclosed (some protection)	☐	☐	☐	
7***. HVAC***// dilution ventilation/ ***present/working***	☐	☐	☐	
8. LEV present, but not appropriate/adequate	☐	☐	☐	
9. No make up air problems(neg press/drafts)	☐	☐	☐	
10. ***Other eng control***	☐	☐	☐	

HUMAN INTERFACE

Human Interface: How likely is it that people will come into contact with the material, or be exposed to it as they do their jobs?
Personal Protective Equipment: To what extent are PPE, work practices, and administrative controls, utilized to decrease the *Human Interface*?

**POTENTIAL**				
HUMAN INTERFACE:				
(Major)	Yes	No	Don’t Know	Describe
1. Manual application of liquid or powder	□	□	□	
2. Manual mix, add, stir chemicals(not ***maint***)	□	□	□	
3. Use of compressed air; ***cleaning*** or process	□	□	□	
4. Employees ***smoke*** at work stations	□	□	□	
5. Ingestion significant route of exposure/MSDS	□	□	□	
6. Work practice contributes to potential	□	□	□	
(Minor)				
7. Dipping parts into liquid (manual)	□	□	□	
8. Heavy workload / Increased metabolic rate	□	□	□	
9. Employees ***eat or drink*** at work station	□	□	□	
10. Contact with work surface contamination	□	□	□	
11. Manual cleaning ***part of the job***	□	□	□	
12. ***Other interface***	□	□	□	
**PROTECTION**				
PERSONAL PROTECTIVE EQUIPMENT:				
(Major)				
1. ***Respirators/dust masks required***	□	□	□	
2***. Protective clothing/equipment req’d: gloves,uniform,glasses,shoes,other:***	□	□	□	
3. Material handling minimized/reduced	□	□	□	
4. Work practice increases protection	□	□	□	
(Minor)				
5. ***Administrative Control procedure in place***	□	□	□	
6. Respirators/dust masks used	□	□	□	
7. Protective clothing ***available/appropriate***	□	□	□	
8. Hand ***cleaning facilities*** nearby	□	□	□	
9. Designated ***eating/break areas used***	□	□	□	
10. Eyewash/shower adequate (bottles?)	□	□	□	
11. ***Other protection***	□	□	□	

Comments:

Additional File 2, published with:
LaMontagne et al (2009): A hazardous substance exposure prevention rating method for intervention needs assessment and effectiveness evaluation: the Small Business Exposure Index. *Environ Health.*
